# Bowel Health, Laxative Use, and Cognitive Function in Older Puerto Rican Adults

**DOI:** 10.1155/jare/2674457

**Published:** 2025-07-24

**Authors:** Deepika Dinesh, Jong Soo Lee, Tammy M. Scott, Katherine L. Tucker, Natalia Palacios

**Affiliations:** ^1^Center for Population Health, University of Massachusetts Lowell, Lowell, Massachusetts, USA; ^2^Department of Public Health, University of Massachusetts Lowell, Lowell, Massachusetts, USA; ^3^Department of Mathematics and Statistics, University of Massachusetts Lowell, Lowell, Massachusetts, USA; ^4^Gerald J. and Dorothy R. Friedman School of Nutrition Science and Policy, Tufts University, Boston, Massachusetts, USA; ^5^Department of Biomedical and Nutritional Sciences, University of Massachusetts Lowell, Lowell, Massachusetts, USA; ^6^Department of Nutrition, Harvard University School of Public Health, Boston, Massachusetts, USA

## Abstract

**Objectives:** Bowel dysfunction and habitual laxative use are indicators of poor gastrointestinal (GI) health and are inversely associated with cognitive function. These associations are understudied in Latino and Hispanic populations. Therefore, we examined the associations between bowel frequency, stool type, laxative use, and cognitive function in the Boston Puerto Rican Health Study (BPRHS).

**Methods:** The BPRHS is a prospective cohort that enrolled 1502 Puerto Rican adults residing in the Boston Area, aged 45–75 years at baseline, with four waves of collection across 13 years. Cognitive function was measured using a composite global cognitive score (GCS) with low scores indicating worse cognitive function. Bowel health at Wave 4 was assessed by self-reported bowel frequency (times/day) and stool type. Self-reported laxative use (yes/no) was assessed at baseline and Wave 4. Adjusting for relevant covariates, we examined cross-sectional association between bowel frequency, stool type and GCS at Wave 4, and serial cross-sectional associations between laxative use and GCS at baseline and Wave 4. We used linear mixed models to examine time-varying laxative use and GCS over 13 years of follow-up.

**Results:** At Wave 4, 155 (27.1%), 235 (41.0%), and 183 (31.9%) participants self-reported normal, low, and high bowel frequency, respectively, and 334 (65.9%), 72 (14.2%), and 101(19.9%) self-reported normal, hard, and loose stool type, respectively. At Wave 4, participants with high bowel frequency had low GCS (*β* = −0.152, *p*=0.02), but stool type was not associated with GCS. Self-reported laxative use increased from 6.8% (baseline) to 18.4% (Wave 4). Laxative users had low GCS at baseline (*β* = −0.13, *p*=0.01) and Wave 4 (*β* = −0.13, *p*=0.05). However, laxative use was not associated with a change in GCS over 13 years of follow-up (laxative use∗time *β* = 0.006, *p*=0.28).

**Conclusion:** Low or high bowel frequency and laxative use may be inversely associated with cognitive function. Our results suggest a relationship between bowel and cognitive health.

**Trial Registration:** ClinicalTrials.gov identifier: NCT01231958

## 1. Introduction

Recent reports estimate that more than 6.7 million people aged 65 years and above in the United States live with dementia [1]. Alzheimer's disease (AD) is the most common type of dementia [1, 2]. The interrelatedness between gastrointestinal (GI) disorders and cognitive dysfunction in neurologic disorders is reported [3–5]. Functional GI disorders (FGID) or disorders of gut–brain interaction are characterized by altered gut motility, such as constipation or diarrhea [6, 7]. Based on a recent survey across 33 countries, the worldwide FGID prevalence is estimated to be as high as 40% [6].

Infrequent bowel frequency, such as constipation, is attributed to age-related loss of enteric neurons [8, 9], variations in diet and fluid intake, sedentary lifestyle, use of medications, metabolic, endocrine, neuropathic, and intestinal disorders, such as irritable bowel syndrome (IBS), and anorectal abnormalities [10]. According to the Rome Foundation, functional constipation includes having fewer than three bowel movements per week, lumpy or hard stool, and a sensation of incomplete evacuation [9, 11]. At the same time, abnormally frequent bowel movements, such as diarrhea, characterized by more than 25% loose watery stool, based on the Rome criteria [11], may be age-related sphincter control loss [9] or GI infections caused by enteric pathogens. These factors may lead to inflammation, loss of sympathetic afferents [12], and frequent bowel movements or diarrhea and loose stool.

Recent studies report on the associations between GI disorders and cognitive function [13], nonamnestic mild cognitive impairment (MCI), AD, and dementia [14–16]. Constipation or hard stools may increase the risk of dementia [17], and a recent review reported that constipation and dementia were positively correlated [18]. GI infections are reported to be associated with both increased dementia risk [19] and diarrhea [20]. Furthermore, dysfunction in specific domains of cognition, such as executive function, attention, and visuospatial memory, are reported across studies among IBS patients [21].

Laxatives, commonly used to manage constipation, are more prevalent with increasing age [22, 23]. In the United States, 40% prevalent laxative use is reported among those with constipation [24]. Furthermore, laxative utilization is used as an estimate of constipation burden [25]. Recently, two studies utilizing UK Biobank data reported associations between laxative use and risk of all-cause dementia, vascular dementia [26], and incident dementia [27], with an interaction between laxative use and genetic susceptibility for dementia [27]. On the other hand, laxative abuse is reported to cause diarrhea [28]. In animal models, osmotic diarrhea induced by over the counter (OTC) laxatives is reported to modulate the gut microbiome [28]. Changes in osmolality and gut microbiome variations by laxative use [28] may lead to a proinflammatory state that may influence cognitive health.

The association between bowel frequency, stool type, laxative use, and cognitive function is underreported, and these associations are understudied in the Hispanic and Latino populations. Therefore, in a prospective cohort of Boston-area Puerto Rican adults, we sought to examine (1) cross-sectional association between self-reported bowel frequency, stool type, and cognitive function; (2) serial cross-sectional associations between self-reported laxative use and cognitive function at two different time points; and (3) the association between laxative use and the trajectory of cognitive function over approximately 13 years of follow-up.

## 2. Methods

### 2.1. Ethics

Study protocols were approved by the Institutional Review Boards (IRBs) at Tufts Medical Center and the University of Massachusetts Lowell (IRB # 17–143; BIDMC IRBs ceded review to UMASS Lowell). Written informed consent was obtained from all participants.

### 2.2. Study Cohort

**Figure 1:**
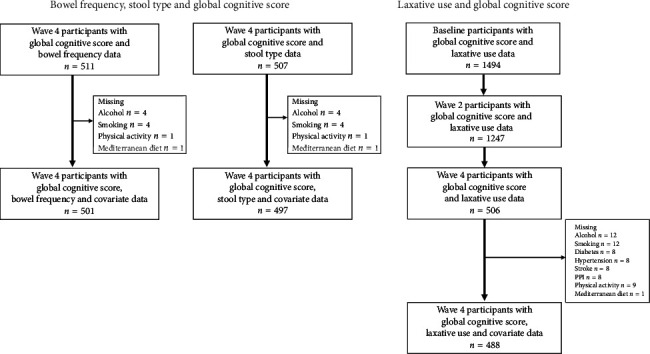
Study design. Participant attrition in the BPRHS, across thirteen years of follow-up over four waves of collection from baseline to Wave 4, is attributed to various reasons including death (*n* = 230), dropping out of the study (*n* = 165), incomplete data (*n* = 15), loss to follow-up (*n* = 190), moving away (*n* = 129), scheduling issues (*n* = 73), poor health (*n* = 34), and missing data due to unknown reasons (*n* = 118). Among 511 and 507 Wave 4 BPRHS participants with complete global cognition score, bowel frequency, and stool type data, respectively, 10 were missing covariates. Among 506 Wave 4 BPRHS participants with complete global cognition score and laxative use data, 18 were missing covariates.

This analysis was conducted with data from the Boston Puerto Rican Health Study (BPRHS), a prospective cohort [29] that enrolled 1502 self-identified Boston-area Puerto Rican adults aged 45–75 years at baseline (2009–2010) and followed through four waves of follow-up (mean 12.7 ± 1.18 years). This is a random sampling of participants recruited from the Boston area based on 2000 census data, and recruitment was done using door-to-door enumeration and community approaches [29]. Baseline participants with low Mini-Mental State Examination (MMSE) score (≤ 10) were excluded from the study [29]. Herein, we present analyses including only participants with complete outcome measure (global cognitive score [GCS]) and exposures, i.e., bowel frequency (*n* = 511 at Wave 4), stool type (*n* = 507 at Wave 4), or laxative use data (*n* = 1494 at baseline, *n* = 1247 at Wave 2, and *n* = 506 at Wave 4) (Figure 1). We used GCSs from baseline, Wave 2, and Wave 4. As reported previously [29], the individual cognitive component scores used to compute the composite GCS were not collected at Wave 3.

### 2.3. Assessment of Cognitive Function

Cognitive tests in Spanish (98%) or English were performed as described previously [29, 30]. The outcome variable, i.e., GCS, was computed as the mean of z-scores for the following tests: MMSE, which is a general measure of cognitive function [31], tests for word list learning [32], recognition, and percentage retention tests to assess verbal memory, digit span forward and backward tests to assess attention and working memory [32], Stroop test involving the naming of color to assess executive function [32], tests for naming as many words as possible starting with a given letter to assess verbal fluency [32], clock drawing [34], and figure copying tests to assess visuospatial function and organization [35]. For participants with incomplete individual test scores that were not missing due to illiteracy, hearing impairment, or poor vision, the scores were imputed using a minimum z-score for the same individual test in the rest of the cohort [29]. The BPRHS team has received permission from Psychological Assessment Resources (https://www.parinc.com) to use the MMSE scale in this research.

### 2.4. Assessment of Bowel Frequency

At Wave 4, the BPRHS bowel health instrument included questionnaires to assess daily and weekly bowel movement frequency, self-reported constipation and diarrhea, stool type based on the Bristol stool scale [36], and laxative use. Participants were asked ‘How often do you usually have bowel movements per day?'; ‘During the past 12 months, how often have you been constipated? (always, most of the time, sometimes, rarely, never, refused, and don't know)'; and ‘During the past 12 months, how often have you had diarrhea? (always, most of the time, sometimes, rarely, never, refused, and don't know).' Participants self-reporting daily bowel frequency of once per day were categorized as healthy (reference group). Low bowel frequency was defined based on two variables, i.e., self-reporting bowel frequency of less than once per day and self-reporting constipation as always or most of the time in the past 12 months. High bowel frequency was also defined based on two variables, i.e., self-reporting bowel frequency of more than once per day and self-reporting diarrhea as always or most of the time in the past 12 months.

### 2.5. Assessment of Stool Type

Self-reported stool type such as hard or loose stools, was based on the Bristol stool scale [36] and ranged from 1–7. Types 1 and 2 were categorized as hard stool, and Types 6 and 7 were categorized as loose stool [1^1^, ^36^].

### 2.6. Assessment of Laxative Use

Laxative use was self-reported and ascertained by careful assessment of medication use by examination of medicine containers during all waves of collection. Laxatives are classified as stimulants, bulk-forming, saline, or osmotic [37]. Laxatives reported in the BPRHS, from baseline to Wave 4, were mainly OTC laxatives, including stimulants such as bisacodyl (diphenylmethane derivatives), oral osmotic such as MiraLAX, oral bulk-forming laxatives such as Metamucil, oral stool softeners, such as Colace, and oral stimulants and rectal suppositories, such as Dulcolax. The use of anthraquinone derivatives such as senna was also self-reported and coded as laxative use. Self-reported laxative use was coded as binary based on use (yes/no) at all waves of collection.

### 2.7. Covariates and Demographic Variables

Data collection was described previously [29]. At each time point, participants provided information on age, sex, education level, and self-reported health conditions [29]. Smoking and alcohol use frequency, history, and type were assessed and categorized based on the current use (yes/no). Physical activity score was based on a modified questionnaire and computed as the sum of hours spent on activities over 24 h and multiplied by weights for the rate of oxygen consumption associated with each activity. Body mass index (BMI) was computed using weight (kg) divided by height (m) squared, and as described and reported previously, BMI of 30 and above was classified as obese. Diabetes was defined as fasting plasma glucose ≥ 126 mg/dL or the use of diabetes medication [29]. Hypertension was defined as blood pressure more than or equal to 140/90 mmHg or the use of hypertension medication. For assessment of Apolipoprotein E (ApoE) status, genomic DNA was isolated from blood (Qiagen, Hilden, Germany) and genotyped to identify genetic polymorphisms (Applied Biosystems TaqMan SNP genotyping system) [29]. Antibiotic use, depression medications, and proton pump inhibitors (PPI) use were self-reported and categorized as yes/no. As described previously [38], PPIs reported in the BPRHS were omeprazole, esomeprazole, lansoprazole, pantoprazole, and rabeprazole. Dietary intake was assessed using a semiquantitative food-frequency questionnaire (FFQ) specifically designed for this population [29]. Mediterranean diet was assessed from the FFQ and categorized (range of 0–9) based on intake of nine dietary components: grains, vegetables, fruits, legumes/pulses/nuts, fish, olive oil, meat/poultry, dairy, and alcohol [29, 33]. Fiber intake was assessed from the FFQ, which included food and supplements (measured in grams). To account for missing data in the categorical variables, such as the ApoE genotype and depression medications, we used the missing indicator method [39]. The average of previous collection waves was used to account for missing data in numeric variables such as BMI, Mediterranean diet, and fiber intake. Covariate selection was based on factors known to influence both the exposure and outcomes and based on those reported in previous studies.

In addition to covariates that were adjusted in regression analyses, as indicated above, we also examined the distribution of relevant variables related to bowel health. Systemic inflammation was assessed using serum C-reactive protein (CRP) (Siemens Medical Solutions Diagnostics, Los Angeles, CA). Comorbidities such as IBS and ulcers were self-reported.

### 2.8. Statistical Analysis

Analyses were performed using R Version 4.2.2 (R Foundation for Statistical Computing). Descriptive statistics based on bowel frequency and laxative users versus nonusers were examined using chi-square or Fisher's tests for categorical variables and Wilcoxon or Kruskal–Wallis tests for continuous variables with normal or non-normal distributions, respectively.

Cross-sectional association between self-reported bowel frequency and GCS was examined at Wave 4 using univariate and multivariable linear regression (MLR) models adjusted for Wave 4 covariates of age, sex, education, BMI, ApoE *ε*4 status, alcohol use, smoking, physical activity score, diabetes, hypertension, stroke, PPI use, depression medications, antibiotic use, Mediterranean diet score, and fiber intake. We also examined the effect modification of the bowel frequency–cognition relationship by fiber intake, laxative use, and physical activity by including a cross-product term for each potential effect modifier and bowel frequency in the fully adjusted regression model.

Cross-sectional association between tertile categories of stool type (hard, normal, and loose) and GCS was examined at Wave 4 in univariate and MLR analyses adjusted for Wave 4 covariates as above.

Serial cross-sectional associations between laxative use (self-reported yes/no) and GCS were performed at baseline and Wave 4 using univariate and MLR models adjusted for baseline or Wave 4 covariates of age, sex, education, BMI, ApoE *ε*4 status, alcohol use, smoking, physical activity score, diabetes, hypertension, stroke, PPI use, depression medications, antibiotic use, and Mediterranean diet score. Because effect modification of genetic susceptibility is suggested in the relationship between laxative use and dementia [27], we used a cross-product term between laxative use and ApoE *ε*4 status in fully adjusted regression models. We further examined the effect modification between laxative use and fiber intake by including cross-product terms in fully adjusted regression models at baseline and Wave 4.

To examine the association between time-varying laxative use (baseline, Wave 2, and Wave 4) and change in GCS over approximately 13 years of follow-up, we used linear mixed-effects models (LMM) (nlme package in R). The global cognitive function included three measures of GCS collected at baseline, Wave 2, and Wave 4 follow-up. Time was a continuous variable. Due to unevenly spaced time points, a continuous Autoregressive 1 correlation structure was used to fit random effects, as reported earlier [38]. Self-reported laxative use, at baseline, Wave 2, and Wave 4 follow-up, and all time-varying covariates (baseline, Wave 2, and Wave 4) were treated as fixed effects. Random effects were modeled for participant study ID (intercept) and time (slope). To examine whether there was a change in GCS based on time-varying laxative use, we added an interaction term between laxative use and time, and the β-coefficient of the interaction term estimates the effect of laxative use on changes in GCS over time. Then, in sensitivity analyses, we performed LMM as above, but restricting to only participants with complete GCSs from baseline to Wave 4.

## 3. Results

### 3.1. Participant Characteristics

At the time of writing, 511 and 507 participants had complete data on GCS (outcome) and bowel frequency and stool type at Wave 4, respectively. A total of 1494, 1247, and 506 participants had complete data on GCS and laxative use at baseline, Wave 2, and Wave 4, respectively (Figure 1).

Among the 511 Wave 4 participants, 139 (27.2%), 210 (41.1%), and 162 (31.7%) self-reported normal, low, and high bowel frequency, respectively (Table 1). In univariate analysis at Wave 4, compared to those with normal bowel frequency, participants with low bowel frequency were more likely to have low physical activity (*p* < 0.01), high prevalence of stroke (*p* < 0.01), and PPI use (*p*=0.02). As expected, they tended to report higher laxative use (*p* < 0.01) and have hard stool (based on Bristol stool Types 1 and 2) (*p* < 0.01) (Table 1). Among 507 Wave 4 participants, 334 (65.9%), 72 (14.2%), and 101(19.9%) self-reported normal, hard, and loose stool type, respectively.

Among 1494 baseline participants, 101 (6.8%) self-reported laxative use. Among 506 Wave 4 participants, 93 (18.4%) self-reported laxative use (Table 1). In univariate analysis at Wave 4, compared to nonusers, laxative users were more likely to use PPIs (*p* ≤ 0.01), more likely to have hard (*p*=0.03) and loose stool (*p*=0.01) (Table 1). Among laxative users, five participants used laxatives continuously from baseline to Wave 4 (approximately 13 years of continuous use), and 34 participants reported using laxatives at two or more waves.

### 3.2. Cross-Sectional Associations Between Bowel Frequency, Stool Type, Laxative Use, and GCS

**Figure 2:**
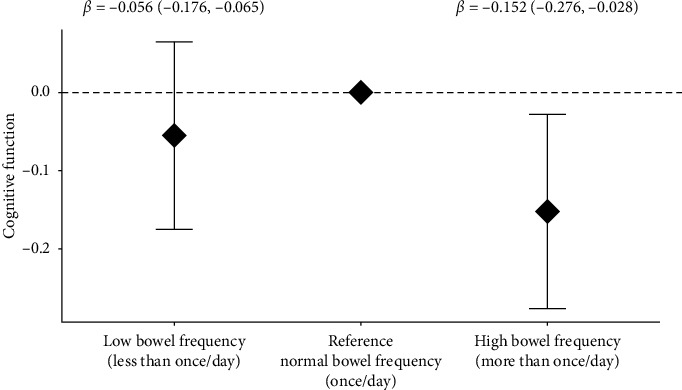
Association between daily bowel frequency and cognitive function at Wave 4. Association between daily bowel frequency and cognitive function at Wave 4 (*n* = 501). Adjusted for age, sex, education, BMI, ApoE and epsi; 4 status, alcohol use, smoking, physical activity score, diabetes, high BP/hypertension, stroke, proton pump inhibitors, depression medications, antibiotic use, Mediterranean diet score, and fiber intake.

At Wave 4, in univariate analyses, we observed an inverse association between high bowel frequency and GCS (*β* = −0.151, 95% CI: −0.293, −0.008, *p*=0.04). After adjusting for Wave 4 covariates of age, sex, education, BMI, ApoE *ε*4 status, alcohol use, smoking, physical activity score, diabetes, hypertension, stroke, PPI use, depression medication and antibiotic use, Mediterranean diet score, and fiber intake, we continued to observe an inverse association between high bowel frequency and GCS (*β* = −0.152, 95% CI: −0.276, −0.028, *P*_adj_=0.02) (Figure 2). Low bowel frequency tended to be inversely associated with GCS in univariate analyses (*β* = −0.144, 95% CI: −0.278, 0.010, *p*=0.04) but not statistically significant after adjusting for relevant covariates as indicated above (*β* = −0.056, 95% CI: −0.176, 0.065, *P*_adj_=0.36) (Figure 2). We did not observe an interaction between bowel frequency and dietary fiber (*P-*int = 0.30), laxative use (*P-*int = 0.53), or physical activity score (*P-*int = 0.48).

At Wave 4, in univariate and multivariable analyses, adjusted for Wave 4 covariates age and sex (Model 1) and additionally adjusted for education, BMI, ApoE *ε*4 status, alcohol use, smoking, physical activity score, diabetes, hypertension, stroke, PPI use, depression medication and antibiotic use, Mediterranean diet score, and fiber intake (model 2), stool type was not associated with GCS (all *p* trend ≥ 0.59) (Supporting Table 1).

**Figure 3:**
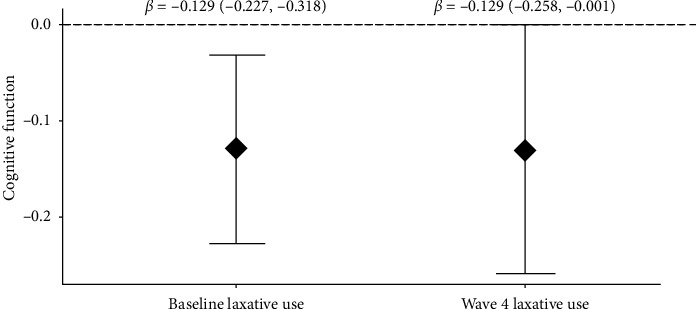
Association between laxative use and cognitive function at baseline and Wave 4. Association between baseline laxative use and baseline cognitive function (*n* = 1459). Adjusted for baseline age, sex, education, BMI, ApoE and epsi; 4 status, alcohol use, smoking, physical activity score, diabetes, high BP/hypertension, stroke, proton pump inhibitors, depression medications, antibiotic use, and Mediterranean diet score. Association between Wave 4 laxative use and Wave 4 cognitive function (*n* = 488). Adjusted for Wave 4 age, sex, education, BMI, ApoE *ε*4 status, alcohol use, smoking, physical activity score, diabetes, high BP/hypertension, stroke, proton pump inhibitors, depression medications, antibiotic use, and Mediterranean diet score.

At baseline, in univariate analysis, baseline laxative use (yes/no) was inversely associated with GCS (*β* = −0.190, 95% CI: −0.305, −0.076, *p* < 0.01). After adjusting for baseline age, sex, education, BMI, ApoE *ε*4 status, alcohol use, smoking, physical activity score, diabetes, hypertension, stroke, PPI use, depression medication and antibiotic use, and Mediterranean diet score, baseline laxative use continued to be inversely associated with GCS (*β* = −0.129; 95% CI: −0.227, −0.318, *P*_adj_=0.01) (Figure 3). There was no interaction between baseline laxative use and baseline dietary fiber intake (*P-*int = 0.39) or ApoE *ε*4 status (*P-*int = 0.78).

We observed a similar trend at Wave 4: in univariate analysis Wave 4 laxative use was inversely associated with Wave 4 GCS (*β* = −0.145; 95% CI: −0.287, −0.002; *p*=0.05). In fully adjusted analyses with Wave 4 covariates, as indicated above, Wave 4 laxative use continued to be inversely associated with Wave 4 GCS (*β* = −0.129, 95% CI: −0.258, −0.001, *P*_adj_=0.05) (Figure 3). There was no interaction between Wave 4 laxative use and Wave 4 fiber intake (*P-*int = 0.77) or ApoE *ε*4 status (*P-*int *p*=0.20).

### 3.3. Time-Varying Laxative Use and Change in GCS

**Figure 4:**
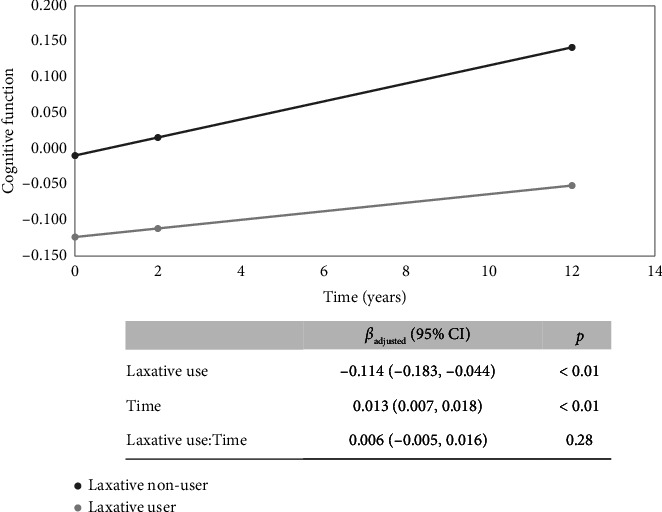
Association between time-varying laxative use and change in cognitive function. Association between time-varying laxative use and change in cognitive function (*n* = 1494). Adjusted for baseline sex, education, and ApoE and epsi; four status and time-varying (baseline, Wave 2, and Wave 4) age, BMI, alcohol use, smoking, physical activity score, diabetes, hypertension, stroke, proton pump inhibitors, depression medications, antibiotic use, and Mediterranean diet score.

In univariate linear mixed models, laxative use was associated with a change in GCS over 13 years of follow-up (laxative use∗time *β* = 0.010, *p*=0.04). However, after adjusting for baseline sex, education, ApoE *ε*4 status, and time-varying covariates of age, BMI, alcohol use, smoking, physical activity score, diabetes, hypertension, stroke, PPI use, depression medication and antibiotic use, and Mediterranean diet score, the association between laxative use with a change in GCS over 13 years of follow-up was attenuated (laxative use∗time *β* = 0.006; *P*_adj_=0.28) (Figure 4).

In sensitivity analyses, restricting to participants with complete GCS from baseline to Wave 4 (*n* = 513), we observed a similar trend, i.e., time-varying laxative use was not associated with a change in GCS over 13 years follow-up (laxative use∗time *β* = 0.002; *P*_adj_=0.78) (Supporting Figure 1). In additional sensitivity analyses, among participants who reported laxative use in two or more waves (*n* = 34), we observed a similar trend, i.e., time-varying laxative use was not associated with a change in GCS over 13 years follow-up (laxative use∗time *β* = 0.0004; *P*_adj_=0.95).

### 3.4. Attrition

We observed 66.3% loss to follow-up among laxative users from baseline to Wave 4, but laxative use did not predict a loss to follow-up (attrition bias) in our study (*p*=0.51).

## 4. Discussion

In this study of older Boston-area Puerto Rican adults, we observed inverse associations between high bowel frequency and cognitive function in a cross-sectional analysis. Low bowel frequency was also inversely associated with cognitive function, although it did not reach statistical significance, likely due to sample size. Our results are supported by a recent study, performed in a predominantly white cohort, that also reports an association between bowel dysfunction and cognitive decline [13], thus providing cross-ethnic validation and supporting the broader applicability of these findings. The association between bowel dysfunction and cognitive decline may be attributed to autonomic dysfunction [40, 41]. GI infections caused by enteric pathogens may contribute to diarrhea by multifactorial mechanisms and effacement of microvilli, which influence water absorption, alterations in ion transport, and tight junctions, which subsequently influence neurotransmitter function and inflammation [20]. Inflammation is associated with loss of the sympathetic afferents [12], which may cause frequent bowel movements or diarrhea. Furthermore, bacterial endotoxins and bacterial amyloids are reported to elicit GI inflammation, which may cause degeneration of enteric neurons and GI dysfunction [12, 42]. Systemic inflammation triggered by gut dysbiosis, and bacterial endotoxins may cause dysfunction in tight junctions and permeability of the GI epithelial barrier, which may subsequently lead to increased permeability of the blood–brain barrier exacerbating neuroinflammation and neurodegeneration in the central nervous system [43].

Studies have reported an increased odds of nonamnestic MCI and dementia among those with constipation [44], low cognitive scores among participants with constipation [45], increased prevalence of AD among those with constipation [15], and a positive correlation between the prevalence and progression of dementia and constipation [18, 46]. Although we observed an inverse association between low bowel frequency and cognitive function, this did not reach statistical significance likely due to a lack of statistical power or differences in the definition of constipation.

While our results suggest an association between bowel frequency and cognitive function, we did not observe an association between stool type and cognitive function, as reported by a previous study [17]. It is worth noting that both low and high bowel frequency were defined using a combination of two self-reported variables, potentially providing a more robust or nuanced measure. In contrast, stool type was based on a single self-reported variable, which may have limited sensitivity in capturing relevant variations. This methodological difference could partly explain the discrepancy in associations observed between these two bowel-related measures and cognitive function.

Next, we examined the association between laxative use and cognitive function. Interestingly, in our cohort, laxative users tended to have an increased prevalence of hard and loose stool (defined by the Bristol stool scale), which suggests laxatives used for constipation or laxative overuse causing loose stool or diarrhea. We also observed an increased prevalence of PPI use among laxative users, suggesting comorbid conditions, and the potential contribution of PPI to gut dysfunction. PPI use may independently be inversely associated with global cognitive function [38]. Therefore, the confounding of PPI use was adjusted in our analyses. Among baseline participants, we observed an inverse association between self-reported laxative use and cognitive function. This trend continued to Wave 4, where we observed a similar pattern of inverse association between laxative use and cognitive function. Our findings, on the inverse association between laxative use and cognitive function in the baseline cohort, are supported by two recent studies conducted using UK Biobank participants, a predominantly White cohort, relating laxative use and risk of all-cause and vascular dementia [26, 27]. Interestingly, both these studies utilized OTC laxatives to define exposure, and our definition of laxative use was also mostly based on OTC laxative medications. Again, our results provide cross-ethnic validation and applying these findings in more diverse clinical settings.

We further examined changes in cognitive function based on time-varying laxative use. Compared to nonusers, laxative users tended to have low cognitive function at baseline and continued to have low cognitive function through 13 years follow-up, but with no significant associations with cognitive function changes over time. Baseline participants who continued to Wave 4 may have better overall health, and this may lead to survivor bias. To address this, we performed a sensitivity analysis only among those participants with complete cognitive function scores from baseline to Wave 4, and we observed a similar trend, i.e., low cognitive scores among laxative users, although not as low as the larger study, but no significant association with a change in cognitive function over time.

Strengths of our study include (1) a comprehensive set of multiwave outcome measures, predictors, and covariates at multiple time points for serial cross-sectional and longitudinal analyses for laxative use; (2) bowel instrument measures to assess bowel frequency and stool phenotypes; (3) availability of comprehensive and relevant covariates to examine these associations in an understudied population of Puerto Ricans, who are reported to have a high prevalence of comorbidities impacting GI and cognitive health; (4) potential confounding factors associated with changes in bowel frequency, such as aging [8, 9, 27], diet, sedentary lifestyle, and use of medications [10], were adjusted in the analyses; and (5) we addressed survivor and selection bias by performing sensitivity analysis restricted to participants with complete cognition data during all waves of collection. We also computed attrition risk and observed that laxative use did not predict a loss to follow-up. Limitations of our study include (1) self-reported bowel frequency, stool type, and laxative use; (2) lack of information on fluid intake which is an important contributor to gut motility; (3) lack of information on cholinergic medications which are reported to be associated with constipation [37], so we could not adjust for these factors in our analyses; and (4) inability to ascertain causality from cross-sectional studies.

In conclusion, we observed an inverse association between laxative use, high bowel frequency, and cognitive function, suggesting that poor bowel health is inversely associated with cognitive function. Furthermore, the gut microbiome may be an important mediator in these associations, and more studies on the association between the gut microbiome, bowel health, and cognitive function are needed.

## Figures and Tables

**Table 1:** Demographics of 511 BPRHS participants.

	Bowel frequency (*n* = 511)				Laxative use (*n* = 506)		
	Normal bowel frequency	Low bowel frequency	High bowel frequency	*p*	Laxative user	Laxative nonuser	*p*
*n* (%)	139 (27.2)	210 (41.1)	162 (31.7)		93 (18.4)	413 (81.6)	
Age median (min, max)	68 (56, 85)	68.5 (55, 88)	68 (55, 87)	0.42^a^	69 (56, 88)	68 (55, 87)	0.57^a^
Female *n* (%)	100 (73)	166 (79)	111 (69)	0.08^c^	72 (80.9)	295 (72.7)	0.14^c^
Alcohol *n* (%)	36 (26.9)	42 (20.2)	44 (27.3)	0.23^c^	16 (11.1)	53 (14.6)	0.37^c^
Smoking *n* (%)	21 (15.4)	29 (14.7)	19 (11.7)	0.64^c^	7 (7.9)	59 (14.6)	0.12^b^
Education ≤ 8th grade *n* (%)	56 (40.9)	108 (52.2)	68 (42.5)	0.07^c^	45 (48.4)	183 (44.3)	0.19^c^
Obese BMI ≥ 30 *n* (%)	64 (51.6)	106 (54.9)	81 (55.1)	0.62^c^	54 (58.1)	234 (56.7)	0.82^c^
Physical activity median (min, max)	27.7 (13.9, 65.5)	26.9 (14.1, 58.9)	27.5 (10.5, 52.8)	< 0.01^a^	26.7 (17.2, 52.8)	27.4 (10.5, 65.5)	0.17^a^
Apolipoprotein E*ε*4 (at least one copy) *n* (%)	30 (21.6)	41 (19.5)	38 (23.4)	0.59^c^	24 (25.8)	86 (20.8)	0.37^c^
Diabetes *n* (%)	66 (47.5)	119 (56.7)	77 (47.5)	0.12^c^	51 (56.7)	204 (50)	0.29^c^
Hypertension *n* (%)	93 (66.9)	156 (74.3)	117 (72.2)	0.32^c^	66 (73.3)	294 (72.1)	0.91^c^
Stroke *n* (%)	4 (2.9)	27 (12.9)	5 (3.1)	< 0.01^b^	8 (8.9)	28 (6.9)	0.50^b^
Depression medication *n* (%)	44 (31.7)	83 (39.5)	64 (39.5)	0.11^c^	35 (37.6)	154 (37.2)	0.89^c^
Proton pump inhibitor use *n* (%)	42 (30.2)	93 (42.3)	56 (34.6)	0.02^c^	51 (56.7)	140 (34.3)	< 0.01^c^
Laxative use *n* (%)	23 (16.5)	107 (50.9)	15 (9.2)	< 0.01^c^			
Hard stool^e^*n* (%)	9 (6.5)	51 (24.3)	12 (7.4)	< 0.01^b^	19 (21.1)	49 (12)	0.03^c^
Loose stool^e^*n* (%)	26 (18.7)	34 (16.2)	41 (25.3)	0.09^b^	27 (30)	72 (17.6)	0.01^c^
Irritable bowel syndrome *n* (%)	4 (2.9)	12 (5.7)	3 (1.9)	0.14^b^	3 (3.3)	16 (3.9)	0.99^b^
Ulcers^f^, *n* (%)	11 (7.9)	20 (9.5)	14 (8.6)	0.89^b^	6 (6.7)	38 (9.3)	0.54^b^
CRP^g^ (mg/L) median (min, max)	2.2 (0.10, 33.8)	2.1 (0.10, 41.8)	3.1 (0.10, 33.4)	0.23^a^	2.4 (0.1, 41.8)	2.5 (0.1, 36.8)	0.29^a^
Mediterranean diet median (min, max)	4 (1, 8)	4 (1, 9)	4 (0, 9)	0.44^a^	4.0 (1, 9)	4.0 (0,9)	0.88^a^
Fiber intake (g) median (min, max)	17.8 (6.8, 68.2)	15.9 (3.29, 49.9)	17.8 (4.3, 47.9)	0.32^a^	19 (6.76, 48.3)	17.7 (3.29, 68.2)	0.45^a^
Global cognitive score (mean ± SD)	0.06 (±0.55)	−0.09 (±0.57)	−0.09 (±0.75)	< 0.01^d^	−0.17 (±0.64)	−0.03 (±0.63)	0.04

*Note:* Demographics for 511 BPRHS participants with complete cognitive function data at Wave 4.

^a^Kruskal–Wallis test.

^b^Fisher's test.

^c^Chi-square test.

^d^Wilcoxon test.

^e^Stool type based on Bristol stool scale: Types 1 and 2 are defined as hard stool and Types 6 and 7 are defined as loose stool.

^f^Ulcers (stomach, duodenal, or peptic).

^g^C-reactive protein (CRP).

## Data Availability

Data are available on request from the authors.

## Supporting Information

Supporting Information 1 Supporting Figure 1: Association between time-varying laxative use and change in cognitive function only among 513 participants with complete cognitive function scores from baseline to Wave 4. Adjusted for baseline sex, education, and ApoE *ε*4 status and time-varying (baseline, Wave 2, and Wave 4) age, BMI, alcohol use, smoking, physical activity score, diabetes, hypertension, stroke, PPIs, depression medications, antibiotic use, and Mediterranean diet score.

Supporting Information 2 Supporting Table 1: Association between categories of stool type and cognitive function at Wave 4. ^a^Adjusted for age and sex (*n* = 507). ^b^Adjusted for age, sex, education, BMI, ApoE *ε*4 status, alcohol use, smoking, physical activity score, diabetes, high BP/hypertension, stroke, proton pump inhibitors, depression medications, antibiotic use, Mediterranean diet score, and fiber intake (*n* = 497). ^c^Using a linear variable representing tertile medians.
