# Supplementary figures and images for: Bowel Health, Laxative Use, and Cognitive Function in Older Puerto Rican Adults
Source: J Aging Res. 2025 Jul 24;2025:2674457. doi: 10.1155/jare/2674457 (PMC12313385; doi:10.1155/jare/2674457)

## Slide 1
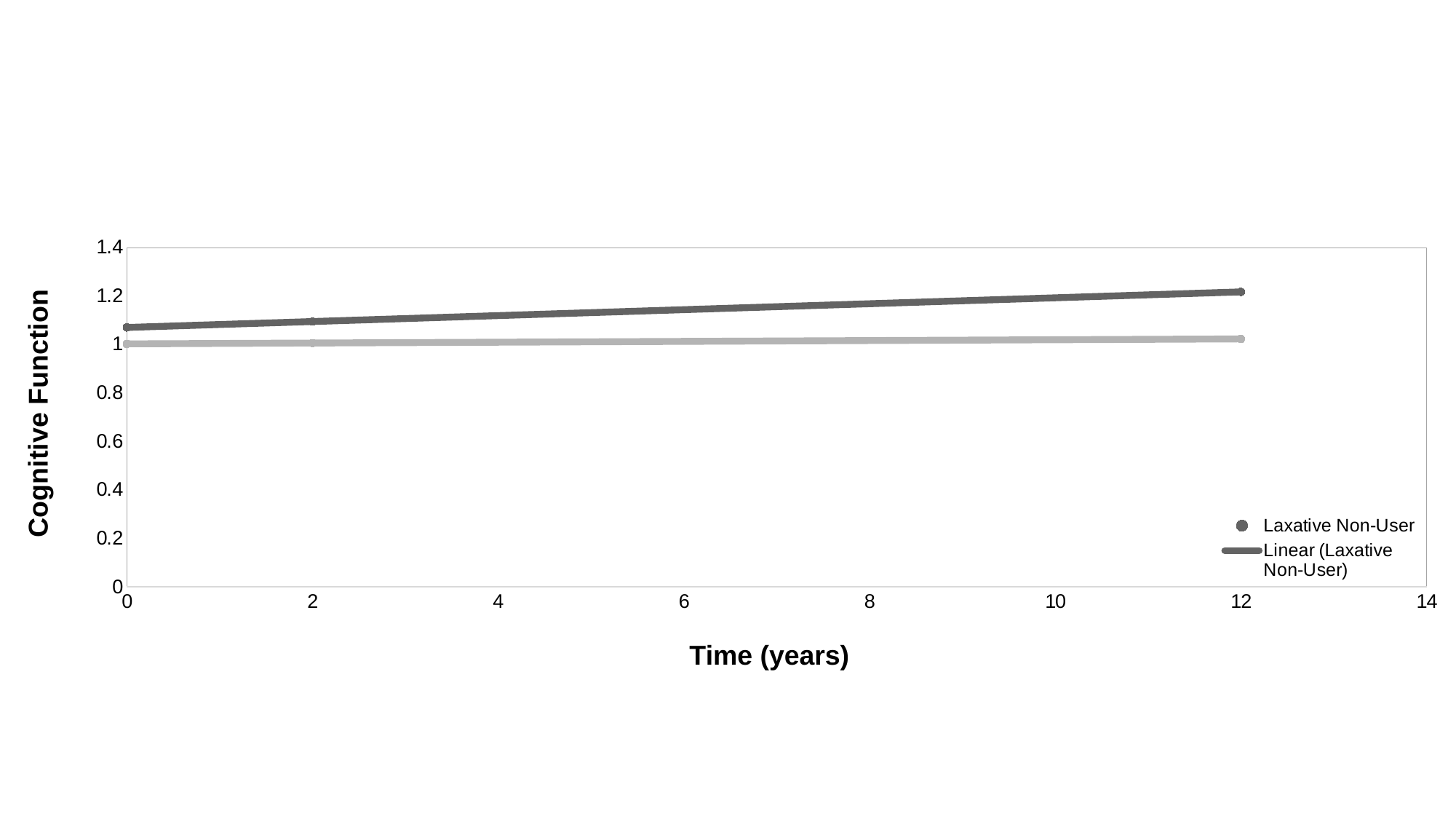

### Chart
| Category | Laxative Non-User | Laxative User |
|---|---|---|Cognitive Function
Time (years)

Supplement: Supporting Information 1 — Supporting Figure 1: Association between time-varying laxative use and change in cognitive function only among 513 participants with complete cognitive function scores from baseline to Wave 4. Adjusted for baseline sex, education, and ApoE ε4 status and time-varying (baseline, Wave 2, and Wave 4) age, BMI, alcohol use, smoking, physical activity score, diabetes, hypertension, stroke, PPIs, depression medications, antibiotic use, and Mediterranean diet score. [file 2674457.f1.pptx]
