# Supplementary material for: Bowel Health, Laxative Use, and Cognitive Function in Older Puerto Rican Adults
Source: J Aging Res. 2025 Jul 24;2025:2674457. doi: 10.1155/jare/2674457 (PMC12313385; doi:10.1155/jare/2674457)
Supplement: Supporting Information 2 — Supporting Table 1: Association between categories of stool type and cognitive function at Wave 4. aAdjusted for age and sex (n = 507). bAdjusted for age, sex, education, BMI, ApoE ε4 status, alcohol use, smoking, physical activity score, diabetes, high BP/hypertension, stroke, proton pump inhibitors, depression medications, antibiotic use, Mediterranean diet score, and fiber intake (n = 497). cUsing a linear variable representing tertile medians. [file 2674457.f2.docx]

### Supplementary Table 1. Association between categories of stool type and cognitive function at wave 4

|  | | Stool type - Hard | Stool type - Normal | Stool type - Loose | *P* trend^c^ |
| --- | --- | --- | --- | --- | --- |
|  | | (Bristol Stool Scale 1-2) | (Bristol Stool Scale 3-5) | (Bristol Stool Scale 6-7) |  |
|  | | β (95% CI) |  | β (95% CI) |  |
| Model 1^a^ | -0.083 (-0.286, 0.033) | | Ref | -0.123 (-0.301, -0.022) | 0.59 |
| Model 2^b^ | | -0.049(-0.192, 0.095) | Ref | -0.060(-0.185, 0.064) | 0.84 |

^a^ Adjusted for age and sex (n=507)
^b^ Adjusted for age, sex, education, BMI, ApoE ε4 status, alcohol use, smoking, physical activity score, diabetes, high BP/hypertension, stroke, proton pump inhibitors, depression medications, antibiotic use, Mediterranean diet score, and fiber intake (n=497)
^c^ Using a linear variable representing tertile medians.
